# Integrated Multi-Omic Characterization of the Detachment Process of Adherent Vero Cells with Animal-Based and Animal-Origin-Free Enzymes

**DOI:** 10.3390/cells11213396

**Published:** 2022-10-27

**Authors:** Shouzhi Yu, Yunchao Huang, Chongyang Wu, Weibin Fu, Hongyang Liang, Chen Chen, Yue Cheng, Yancen Guo, Ying Zhang, Hui Wang, Xiaoming Yang

**Affiliations:** 1 Beijing Institute of Biological Products Company Limited, Beijing 100176, China; 2 China National Biotec Group Company Limited, Beijing 100024, China

**Keywords:** cell detachment, cell culture, transcriptomics, proteomics, metabolomics

## Abstract

Cell detachment techniques using animal-derived enzymes are necessary for the production of biopharmaceuticals that are made with the help of adherent cell cultures, although the majority of protein therapeutics (>USD 100 billion of income per year) are made under suspension cultures that do not require animal-derived proteins for manufacture. In this study, we establish the optimal Vero cell detachment process, and analyze physiological changes during cell detachment at the cellular and molecular levels. Using flow cytometry, we find that animal-based enzymes are more likely to induce apoptosis than animal-origin-free enzymes. We analyze the levels of RNAs, proteins, and metabolites in cells treated with two detachment strategies, and identify 1237 differentially expressed genes, 2883 differential proteins, and 210 differential metabolites. Transcriptomic analysis shows that animal-origin-free enzymes have a less significant effect on gene expression levels. Combined with proteomic analysis, animal-based enzymes affect the oxidative phosphorylation process and reduce the mRNA and protein levels of Cytochrome C Oxidase Assembly Protein 17 (COX17), which is a Cytochrome C Oxidase Copper Chaperone involved in the mitochondrial respiratory chain. Metabolomics analysis indicates that the levels of spermine and spermidine, which are involved in the glutathione metabolism pathway and apoptosis inhibition, are significantly reduced. Therefore, COX17, spermine, and spermidine may be biomarkers for evaluating the cell subculture process. In conclusion, we have deeply characterized the cell subculture process through multi-omics, which may provide important guidance for research and process evaluation to optimize cell detachment processes.

## 1. Introduction

In the manufacture of numerous biological drugs, a large amount of cell-culture-based biomass is required for each batch of the final product, and for some of these cultures, the detachment of cells adherent to glass, plastic or microcarrier surfaces and the type of subcultivation technology is an important process factor to generate that large biomass [1]. For those latter types of manufacturing, from the working bank to the final generation, the cells need to be detached and passaged many times to reach the scale of production for the target product, such as the SARS-CoV-2 vaccine [2], inactivated polio vaccine (IPV), oral polio vaccine (OPV), or rabies vaccines [1,3]. Different detachment processes will not only affect the number of cells [4], but, more importantly, the quality of cells (such as the physiological function [4,5,6], and metabolic status [4,7], etc.), and also will directly affect the yield and quality of target products, such as viruses and proteins.

Traditional indexes for evaluating digestive performance include cell density, cell viability, and glucose metabolism rate [8], etc. Although these indexes play an important role in guiding cell passage and industrial production, they cannot accurately and systematically describe the physiological state of cells in order to guide the establishment and optimization of digestive process.

Multi-omics technology refers to the modern biology research system in a series of high-throughput analyses based on testing technology research methods, including metabolomics, proteomics, genomics, and proteomics [9], etc., which can help to determine strategies, such as the best growth environment for Vero cells, and virus production under high cell density [1]. For example, proteomics was used to analyze rabies virus replication in Vero cells under two different media conditions [10], and quantitative proteomics revealed changes in the response of Vero cells to the porcine epidemic diarrhea virus [11].

Vero is a cell line approved by the World Health Organization for production and is widely used in the production of biological drugs, such as inactivated COVID-19 vaccines [2], poliomyelitis inactivated vaccines [12], rabies vaccines [3], yellow fever vaccines [13], enterovirus 71 vaccines [14], smallpox vaccines [15], and other traditional vaccines or biological drugs [16]. To date, more than five billion doses of inactivated COVID-19 vaccines have been produced using Vero cells as substrates [17]. In addition, Vero cells are widely used to study the molecular mechanisms of virus infection [18,19].

The process of cell detachment is affected by many factors, such as temperature, procedure, and time. The most important factor is the type of digestive solution. There are two types of digestive solutions commonly used in the production of biological products: biogenic and abiogenic. Animal-based enzymes are represented by trypsin, which can hydrolyze proteins between cells and disperse cells. Animal-based enzymes can be obtained in large quantities in the pancreas and are easy to purify, so they are widely used in various cell culture processes [20]. However, animal-based enzymes comprise animal-derived cell digestive enzymes, which can be contaminated by mammalian pathogens and encounter many other problems related to animal-derived substances [21]. Some studies have shown that the use of animal-based enzymes to dissociate adherent cells from the culture substrate may cleave proteins from the cell membrane, resulting in the failure of some cell functions [4]. Huang et al. found that the expression of 36 proteins in MCF-7 cells changed after trypsin treatment [4]. Analysis of the altered proteins revealed that many differentially expressed proteins were involved in the chaperone function (e.g., HSP-60, HSP-90β, and protein disulfide isomerase A3), suggesting that animal-based enzyme detachment may induce stress responses in subcultured cells. In addition, animal-based enzymes were found to decrease the expression levels of proteins related to growth and metabolism, and to increase the expression levels of proteins related to apoptosis. Liu et al. found that the use of trypsin detachment could also affect the autoimmune function of primary cells [22].

In recent years, as an alternative to traditional animal-based enzymes, animal-origin-free enzymes have been gradually applied [23,24,25]; they can cleave peptide bonds at the C-terminus of lysine and arginine, and can directly replace animal-based enzymes. Animal-origin-free enzymes are stable at room temperature and have mild effects on the cells. They can be used to digest mammalian cells in various adhesion states, including CHO, Vero, HEK293, A529, keratinocyte, and embryonic stem cells. The high specificity of non-animal-derived enzymes can reduce the possible damage to cells caused by other enzymes present in some animal-based enzyme extracts, such as trypsin [4,26,27,28], and lysozymes [29], etc.

In the present study, we established the optimal detachment process for Vero cells, used multi-omics research technology to deeply analyze the physiological state of Vero cells after detachment, and found biomarkers that may be used to evaluate the cell detachment process. Our investigations provide new tools and ideas for the continuous optimization of cell detachment technology, and has a certain guiding significance for the industrial production of biological drugs.

## 2. Materials and Methods

### 2.1. Cell Lines and Cell Cultures

Vero cells were purchased from the UK Health Service (Biological Collection). Vero cells were cultured in Minimum Essential Medium (MEM) (Gibco, New York, NY, USA) supplemented with 10% fetal bovine serum at 37 °C in a humidified atmosphere with 5% CO_2_, and passaged when the cells were cultured to 90% density. Trypsin is an example of an animal-based enzyme, while TrypLE is an example of an animal-origin-free enzyme. The 0.25% EDTA-Trypsin was provided by vaccine room six of the Beijing Institute of Biological Products Co., Ltd. (Beijing, China). TrypLE was 1× and purchased from Gibco (Brooklyn, NY, USA), and contains 200 mg/L KCl, 200 mg/L KH_2_PO_4_, 8000 mg/L NaCl, 2160 mg/L Na_2_HPO_4_-7H_2_O, 457.6 mg/L EDTA, and 100 mg/L Phenol Red. Non-enzymatic cell lysate was purchased from Sciencell Company (Carlsbad, CA, USA), and the product number was 0123. Vero cell growth medium containing 10% bovine serum was provided by vaccine room six of the Beijing Institute of Biological Products Co., Ltd. Newborn bovine serum was purchased from Zhejiang Tianhang Biotechnology Co., Ltd. (Zhejiang, China).

### 2.2. Cell Detachment Processes

Vero cells were plated into 24-well plates at a concentration of 2 × 10^5^/well, and after 12–24 h, we incubated trypsin at 25 °C, trypsin at 37 °C, TrypLE detachment solution at 25 °C, and TrypLE detachment solution at 37 °C. Detachment took place at 37 °C with four detachment times of 5 min, 10 min, 15 min, and 20 min. After detachment, we used a growth solution containing 10% serum to terminate the detachment reaction or directly discarded the detachment solution without stopping the detachment reaction. After the cells were treated with the abovementioned detachment processes, the cell viability, cell aggregation rate, and cell metabolism were detected.

### 2.3. Cell Number, Cell Viability, and Cell Aggregation Rate Analysis

An IC1000 Countstar automatic cell counter and cell counting plates were purchased from Shanghai Ruiyu Biotechnology Co., Ltd. (Shanghai, China). Vero cells were detached with the corresponding detachment conditions: 20 µL of the cell suspension was aspirated, added to the cell counting plate, and the number of cells, cell viability, and cell aggregation rate were detected using the Countstar automatic cell counter.

### 2.4. Cell Growth and Proliferation Assay

Vero cells were plated into six-well plates, with 5 × 10^5^ cells per well. After 12–24 h, Vero cells were detached with trypsin or TrypLE detachment solution at 25 °C or 37 °C, and then the cells were counted and plated into 96-well plates. Five thousand cells were plated in each well, and after culturing in a 96-well plate for 24, 48, 72, and 96 h, the number of cells was detected, and the cell growth curve was drawn.

### 2.5. Cell Glucose Metabolism Analysis

Before cell detachment, we used an SBA-40E biosensor analyzer to detect the glucose content in the cell culture medium before detachment. After the cells were detached with the corresponding detachment conditions, we continued to culture them in a petri dish for 1 h; then the cell culture medium was removed, and the glucose content in the culture medium was detected after detachment. The difference in glucose content before and after detachment was calculated, denoting the change in cellular glucose metabolism caused by the detachment process.

### 2.6. Apoptosis Analysis

An annexin V-FITC/PI apoptosis detection kit was purchased from Jiangsu Jikai Biotechnology Co., Ltd. (Jiangsu, China); the product number was KGA108. We detached the Vero cells, washed the cells twice with PBS (centrifugation at 2000 rpm for 5 min), and collected 1–5 × 10^5^ cells. We added 500 µL of binding buffer to suspend the cells and 5 µL of Annexin V-FITC, mixed the solution well, then added 5 µL of PI and mixed well. The mixture was allowed to react in the dark at room temperature for 5–15 min. We reserved three groups of Vero cell samples for each experiment, namely, the Vero cell control, FITC single-staining control, and PI single-staining control. During cytometry detection, the boundary between apoptotic cells and normal cells was set, and FITC and PerCP channels were selected for observation and detection using flow cytometry.

### 2.7. Sample Grouping and Processing for Multi-Omic Analysis

**Figure 1:**
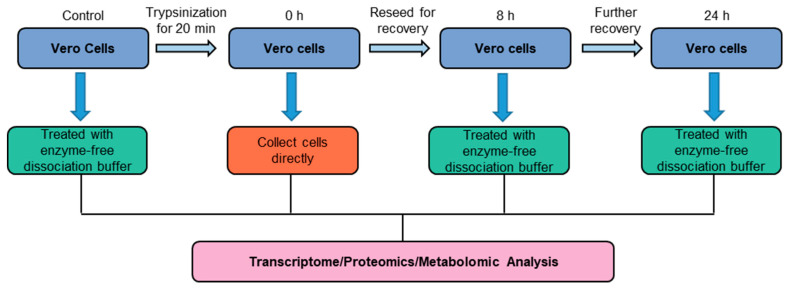
Overview of the workflow of the cell detachment process.

Transcriptomic, metabolomic, and proteomic analyses were undertaken for the cells treated with different detachment processes. The Vero cells were divided into three groups. The first group (Group 1) was detached with trypsin at 37 °C. The cells collected immediately after detachment were set to 0 h, and the detached cells were placed back into the cell incubator for 8 h. After the cells were detached with a nonenzymatic detachment solution and collected at the 8 h timepoint, the cells after trypsinization at 37 °C were further cultured for 24 h, and the cells were detached with a nonenzymatic detachment solution and collected at the 24 h timepoint. The second group (Group 2) was detached with TrypLE at 37 °C, then we set the cells collected immediately after detachment as 0 h, placed the detached cells back into the cell incubator for 8 h, and detached the recovered cells with a nonenzyme detachment solution. After 8 h, we continued to culture the cells detached with TrypLE detachment solution at 37 °C for 24 h, and then detached the cells with a nonenzyme detachment solution at the 24 h timepoint. For the third group (Group 3), we used a 4 °C TrypLE detachment solution at 25 °C detachment; the cells collected immediately after detachment were set to 0 h, and the detached cells were placed back into the cell incubator for 8 h. The cells collected after detachment with the nonenzyme detachment solution were set to 8 h, and detached with TrypLE detachment solution at 4 °C. After the cells were cultured for 24 h, the collected cells were detached with a nonenzymatic detachment solution and set to 24 h. In addition, Vero cells were detached with an enzyme-free detachment solution at 37 °C, and the immediately harvested cells were set as the control group. The experimental process and group settings are shown in Figure 1.

### 2.8. RNA-seq

RNA-seq was performed on three biological replicates for each sample at 0 h, 8 h, and 24 h after treatment with three kinds of detachment solutions (a total of 27 samples). The total RNA of Vero cell samples under different detachment conditions was extracted using Trizol reagent. RNA integrity was assessed using the RNA Nano 6000 Assay Kit of the Bioanalyzer 2100 system (Agilent Technologies, Santa Clara, CA, USA). Total RNA was used as input material for the RNA sample preparations. Briefly, mRNA was purified from total RNA using poly-T oligo-attached magnetic beads. Fragmentation was carried out using divalent cations under elevated temperatures in a First-Strand Synthesis Reaction Buffer (5×). First-strand cDNA was synthesized using a random hexamer primer and M-MuLV Reverse Transcriptase (RNase H−). Second-strand cDNA synthesis was subsequently performed using DNA Polymerase I and RNase H. Remaining overhangs were converted into blunt ends via exonuclease/polymerase activities. After adenylation of the 3′ ends of DNA fragments, the adaptors with a hairpin loop structure were ligated to prepare for hybridization. In order to select cDNA fragments of 370–420 bp in length, the library fragments were purified using an AMPure XP system (Beckman Coulter, Beverly, CA, USA). Then, PCR was performed with Phusion High-Fidelity DNA polymerase, Universal PCR primers, and Index (X) Primer. Finally, PCR products were purified (AMPure XP system) and the library quality was assessed on the Agilent Bioanalyzer 2100 system. The clustering of the index-coded samples was performed on a cBot Cluster Generation System using TruSeq PE Cluster Kit v3-cBot-HS (Illumina, San Diego, CA, USA) according to the manufacturer’s instructions. After cluster generation, the library preparations were sequenced on an Illumina Novaseq platform and 150 bp paired-end reads were generated. Differential expression analysis of two conditions/groups (two biological replicates per condition) was performed using the DESeq2 R package (1.20.0). DESeq2 provides statistical routines for determining differential expression in digital gene expression data using a model based on the negative binomial distribution. The resulting *p*-values were adjusted using Benjamini and Hochberg’s approach for controlling the false discovery rate. Genes with an adjusted *p*-value ≤ 0.05 found by DESeq2 were assigned as differentially expressed. Gene Ontology (GO) enrichment analysis of differentially expressed genes was implemented using the clusterProfiler R package, in which gene length bias was corrected. GO terms with a corrected *p*-value of less than 0.05 were considered significantly enriched by differentially expressed genes. KEGG is a database resource used for understanding the high-level functions and utilities of biological systems, such as the cell, the organism, and the ecosystem, from molecular-level information, especially large-scale molecular datasets generated by genome sequencing and other high-throughput experimental technologies (http://www.genome.jp/kegg/ (accessed on 26 May 2022)). We used the clusterProfiler R package (version 3.8.1) to test the statistical enrichment of differential expression genes in KEGG pathways.

### 2.9. Proteomics

Proteomics was performed on three biological replicates for each sample. Vero cells were lysed with a DB lysis buffer (8 M Urea, 100 mM TEAB, pH 8.5), followed by 5 min of ultrasonication on ice. The lysate was centrifuged at 12,000× *g* for 15 min at 4 °C and the supernatant was reduced with 10 mM DTT for 1 h at 56 °C and subsequently alkylated with sufficient iodoacetamide for 1 h at room temperature in the dark. Following a BCA, normalized quantities of protein were detached overnight with trypsin. The resulting peptides were fractionated using a C18 column (Waters BEH C18, 4.6 × 250 mm, 5 μm) on a Rigol L3000 HPLC system, with the column oven set to 45 °C. The eluates were monitored at UV 214 nm, collected, and finally combined into six fractions. All fractions were dried under a vacuum, and then reconstituted in 0.1% (*v*/*v*) formic acid (FA) in water. For transition library construction, shotgun proteomics analyses were performed using an EASY-nLCTM 1200 UHPLC system (Thermo Fisher, Bremen, Germany) coupled with a Q Exactive^TM^ HF-X mass spectrometer (Thermo Fisher) operating in the data-dependent acquisition (DDA) mode. A half-sample containing 4 μg of fraction supernatant and 0.8 μL of iRT reagent was injected into a homemade C18 Nano-Trap column (4.5 cm × 75 μm, 3 μm). Peptides were separated in a homemade analytical column (15 cm × 150 μm, 1.9 μm). The separated peptides were analyzed using a Q ExactiveTM HF-X mass spectrometer (Thermo Fisher), with a Nanospray Flex™ (ESI) ion source, spray voltage of 2.1 kV, and ion transport capillary temperature of 320 °C. The full scan range was from m/z 350 to 1500 with a resolution of 120,000 (at m/z 200), the automatic gain control (AGC) target value was 3 × 10^6^, and the maximum ion injection time was 80 ms. The top 40 precursors of the highest abundance in the full scan were selected and fragmented using higher energy collisional dissociation (HCD) and analyzed in MS/MS, where the resolution was 15,000 (at m/z 200), the automatic gain control (AGC) target value was 5 × 10^4^, the maximum ion injection time was 45 ms, the normalized collision energy was 27%, the intensity threshold was 1.1 × 10^4^, and the dynamic exclusion parameter was 20 s.

The raw data of MS detection were used to construct a DDA spectrum library. Mobile phases A (0.1% FA in H_2_O) and B (0.1% FA in 80% ACN) were used to develop a gradient elution. A half-sample containing 4 μg fraction supernatant and 0.8 μL iRT reagent was injected into an EASY-nLCTM 1200 UHPLC system (Thermo Fisher) coupled with an Orbitrap Q ExactiveTM HF-X mass spectrometer (Thermo Fisher) operating in the data-independent acquisition (DIA) mode with a spray voltage of 2.1 kV, Nanospray Flex™ (ESI), and capillary temperature of 320 °C. For DIA acquisition, the m/z range was from 350 to 1500. The MS1 resolution was set to 60,000 (at m/z 200), the full scan AGC target value was 5 × 10^5^, and the maximum ion injection time was 20 ms. Peptides were fragmented by HCD in MS2, in which the resolution was set to 30,000 (at 200 m/z), the AGC target value was 1 × 10^6^, and the normalized collision energy was 27%. The search and identification results obtained using Proteome Discoverer software (version 2.2, Thermo Fisher, Bremen, Germany) were imported into Spectronaut (version 14.0, Biognosys) software to generate a library. Eligible peptides and product ions were selected from the spectrum by setting peptides and ion pair selection rules [30] to generate a Target List. The DIA data were imported, and ion-pair chromatographic peaks were extracted according to the Target List. We matched the ion and calculating peak area to achieve the qualitative and quantitative assessment of peptides. The iRT was added to the sample for correcting retention time, and the precursor ion Qvalue cutoff was set to 0.01. The protein quantitation results were statistically analyzed using a *t*-test.

### 2.10. Metabolomics

Metabolomics was performed on six biological replicates for each sample. The samples were resuspended with prechilled 80% methanol by well vortex. Then, the samples were melted on ice and whirled for 30 s. After sonification for 6 min, they were centrifuged at 5000 rpm and 4 °C for 1 min. The supernatant was freeze-dried and dissolved with 10% methanol. Finally, the solution was injected into the LC-MS/MS system for analysis [31,32]. UHPLC-MS/MS analyses were performed using a Vanquish UHPLC system (Thermo Fisher, Bremen, Germany) coupled with an Orbitrap Q ExactiveTM HF mass spectrometer (Thermo Fisher, Bremen, Germany) at Novogene Co., Ltd. (Beijing, China). Samples were injected onto a Hypersil Gold column (100 × 2.1 mm, 1.9 μm) using a 12 min linear gradient at a flow rate of 0.2 mL/min. The eluents for the positive polarity mode were eluent A (0.1% FA in water) and eluent B (methanol). The eluents for the negative polarity mode were eluent A (5 mM ammonium acetate, pH 9.0) and eluent B (methanol). The solvent gradient was set as follows: 2% B, 1.5 min; 2–85% B, 3 min; 85–100% B, 10 min; 100–2% B, 10.1 min; and 2% B, 12 min. A Q ExactiveTM HF mass spectrometer was operated in positive/negative polarity mode with a spray voltage of 3.5 kV, capillary temperature of 320 °C, sheath gas flow rate of 35 psi, aux gas flow rate of 10 L/min, S-lens RF level of 60, and aux gas heater temperature of 350 °C. The raw data files generated by UHPLC-MS/MS were processed using Compound Discoverer 3.1 (CD3.1, Thermo Fisher) to perform peak alignment, peak picking, and quantitation for each metabolite. The main parameters were set as follows: retention time tolerance, 0.2 min; actual mass tolerance, 5 ppm; signal intensity tolerance, 30%; signal/noise ratio, 3; and minimum intensity. After that, peak intensities were normalized to the total spectral intensity. The normalized data were used to predict the molecular formula based on additive ions, molecular ion peaks, and fragment ions. Then peaks were matched with the mzCloud (https://www.mzcloud.org/ (accessed on 24 May 2022)), mzVault, and MassList database to obtain the accurate qualitative and relative quantitative results. Statistical analyses were performed using the statistical software R (R version R 3.4.3, FSF, Boston, MA, USA), Python (Python 2.7.6 version, PSF, Wilmington, DE, USA), and CentOS (CentOS release 6.6, Red Hat, Raleigh, NC, USA); when data were not normally distributed, normal transformations were attempted using the area normalization method. These metabolites were annotated using the KEGG database (https://www.genome.jp/kegg/pathway.html (accessed on 10 Febrary 2018)), HMDB (https://hmdb.ca/metabolites, accessed on 23 Febrary 2022), and LIPIDMaps databases (http://www.lipidmaps.org/ (accessed on 5 November 2021)). Principal component analysis (PCA) and partial least-squares discriminant analysis (PLS-DA) were performed in metaX (version 2.0, BGI, Shenzhen, China) [33] (a flexible and comprehensive software package for processing metabolomics data). We applied univariate analysis (*t*-test) to calculate the statistical significance (*p*-value). Metabolites with VIP > 1, *p*-value < 0.05, and fold change ≥ 2 or FC ≤ 0.5 were considered to be differential metabolites. Volcano plots were used to filter metabolites of interest based on log2 (FoldChange) and –log10 (*p*-value) of metabolites by ggplot2 in R language. For clustering heat maps, the data were normalized using z-scores of the intensity areas of differential metabolites and were plotted using the Pheatmap package in R language (FSF, Boston, MA, USA). The functions of these metabolites and metabolic pathways were studied using the KEGG database. The metabolic pathway enrichment of differential metabolites was performed; when the ratios were satisfied by x/n > y/N, the metabolic pathways were considered enriched, and when the *p*-value of the metabolic pathway was <0.05, the metabolic pathway was considered to have experienced statistically significant enrichment.

### 2.11. Combined Transcriptome and Metabolome Analysis

Based on the results of DEM and DEG analyses, those corresponding to the same treatment were simultaneously mapped to the KEGG pathway to elucidate the relationship between the genes and the metabolites. Based on the results of DEM and DEG enrichment analyses, histograms were drawn to highlight the relative differences in metabolite and gene pathway enrichment. Correlation analysis was performed for the genes and metabolites detected in each differential subgroup. Pearson’s correlation coefficients for the genes and the metabolites were calculated using the COR program of R, and the differential multiplicity of gene metabolites was shown using a nine-quadrant plot, with Pearson’s correlation coefficient >0.8 in each differential subgroup. All DEMs and DEGs were selected to construct O2PLS (bidirectional orthogonal projection of latent structures) models. Variables in different datasets with different correlations and weights were initially identified by loading plots to filter out important variables affecting other omics [34].

## 3. Results

### 3.1. Effects of Different Detachment Processes on Cell Apoptosis

**Figure 2:**
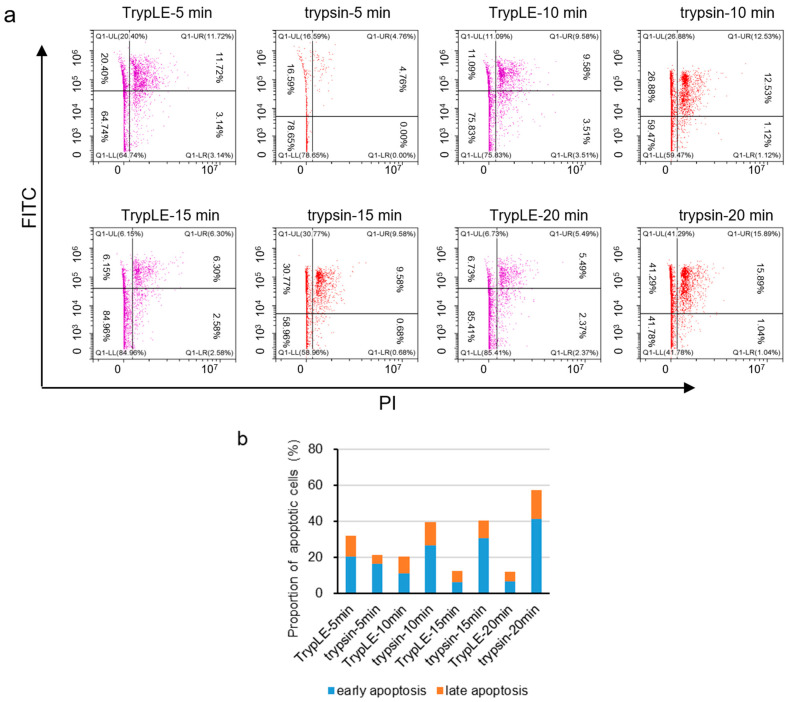
Animal-based enzymes promote cell apoptosis compared with animal-origin-free enzymes. (**a**) Animal-based enzymes and animal-origin-free enzymes at 37 °C resulted in apoptosis. A total of 5 × 10^5^ cells were plated in each well of a six-well plate, and, after culturing for 12–24 h, the cells were detached with animal-based enzymes (trypsin) or animal-origin-free enzymes (TrypLE) at 37 °C for 5 min, 10 min, 15 min, or 20 min, and FITC (5-Fluoresceinisothiocyanate)/PI (Propidium Iodide) dual-color labeling flow cytometry was performed. Cells stained only with FITC represent early apoptotic cells, and cells stained only with PI represent cells with late apoptosis. (**b**) Quantification column chart of Figure 2a.

In order to explore the effects of animal-based enzymes and animal-origin-free enzymes on the physiological state of cells, we detached the cells using different detachment processes (see Section 2.2. for specific conditions), and detected the effects on cell viability, aggregation rate, cell growth and proliferation, glucose metabolism, and apoptosis. Firstly, we found that different detachment processes had no significant effect on cell viability (Supplementary Figure S1a), aggregation rate (Figure S1b), cell growth and proliferation (Figure S1c), or glucose metabolism (Figure S1d). Next, we examined the effect of different detachment processes on apoptosis. Vero cells were detached with animal-based enzymes or animal-origin-free enzymes at 37 °C, and then the cells were stained with PI/FITC, and the proportion of apoptotic cells was detected using flow cytometry. The results showed that, after 10 min, 15 min, and 20 min of digesting cells with animal-based enzymes, the proportion of apoptosis was significantly higher than that in the corresponding group of animal-origin-free enzymes (Figure 2a,b), independent of the temperature of the detachment solution (Figure S2). To sum up, in the process of cell detachment, animal-based enzymes were more likely to lead to cell apoptosis than animal-origin-free enzymes.

### 3.2. Transcriptomic Analysis of Different Detachment Processes

**Figure 3:**
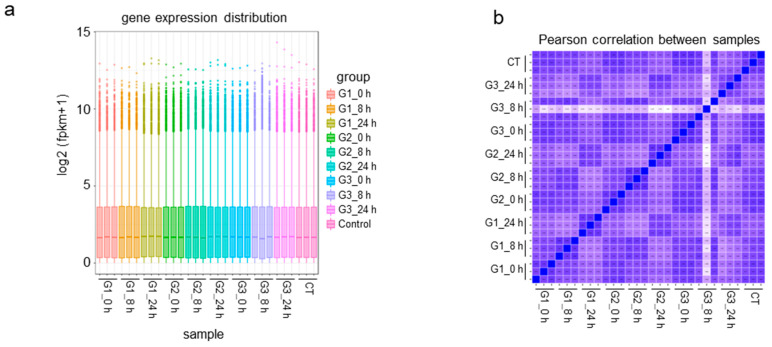

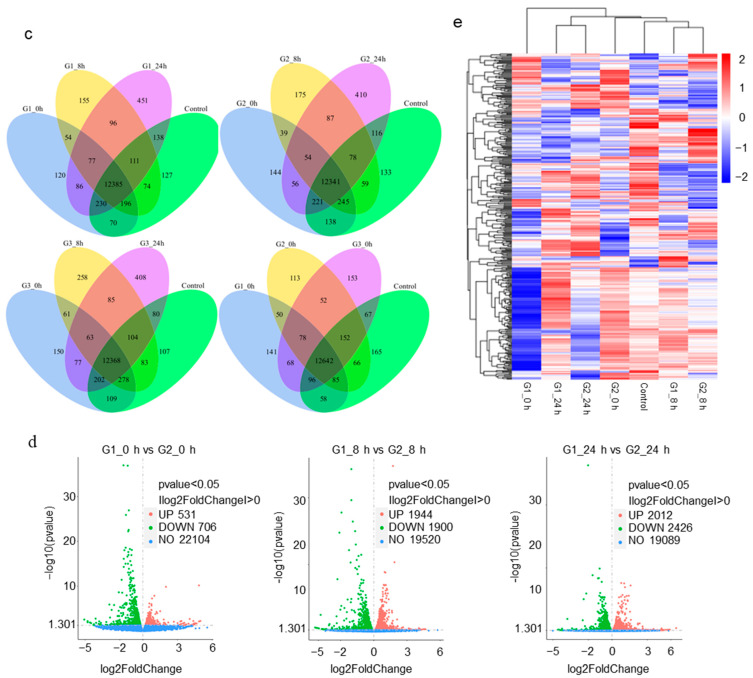

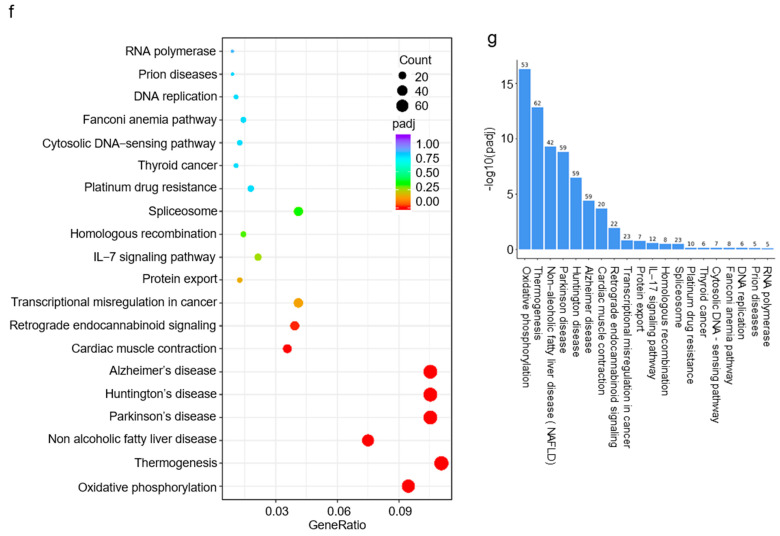
Transcriptomic analysis of animal-based enzymes and animal-origin-free enzymes. (**a**) Gene expression box plot. The abscissa represents different samples, the ordinate is log2 (FPKM + 1), and the box plot of each area is for five statistical measurements (from top to bottom: the maximum value, the upper quartile, the median, the lower four quantile, and the minimum). This figure shows the distribution of gene expression levels in different groups of samples (three biological replicates per group). G1 represents Group 1, which comprises 37 °C animal-based enzyme detachment of cells at 37 °C; G2 represents Group 2, which comprises 37 °C animal-origin-free enzymes to digest cells at 37 °C; G3 represents Group 3, which comprises 4 °C animal-origin-free enzymes being used to digest cells at room temperature; 0 h, 8 h, and 24 h are the number of hours after digesting cells in the above three conditions of detachment solution; the control group represents cells detached at 37 °C with a 37 °C nonenzymatic detachment solution. (**b**) A heatmap of correlations between the samples. The horizontal and vertical coordinates represent different samples. R2 represents the correlation between two samples, and the closer R2 is to 1, the stronger the correlation. (**c**) Venn diagram of gene co-expression between samples. (**Top left**) G1 represents Group 1, which means that Vero cells were detached with animal-based enzymes at 37 °C 0 h, 8 h, and 24 h after digesting the cells; the control group represents cells detached with a 37 °C nonenzyme detachment solution at 37 °C. (**Top right**) G2 represents Group 2, and Vero cells were detached at 37 °C with animal-origin-free enzymes at 37 °C. (**Bottom left**) G3 represents Group 3, and Vero cells were detached with 4 °C animal-origin-free enzymes at room temperature. (**Bottom right**) Venn diagram of the co-expression of G1_0h, G2_0h, G3_0h, and the control group. (**d**) Volcano plot of differential gene expression at 0 h, 8 h, and 24 h (from left to right) after the cells were detached with animal-based enzymes and animal-origin-free enzymes at 37 °C. G1 represents Group 1, which comprises 37 °C animal-based enzymes that detached Vero cells at 37 °C; G2 represents Group 2, which comprises 37 °C animal-origin-free enzymes used to digest Vero cells at 37 °C; 0 h, 8 h, and 24 h are the detachment solutions using the above two conditions a certain number of hours after the detachment of cells. Red represents genes upregulated in the animal-based enzymes group compared with animal-origin-free enzymes; green represents genes downregulated in the animal-based enzymes group compared with animal-origin-free enzymes; blue represents genes with no significant difference between the two groups. (**e**) Cluster heatmap. The horizontal axis represents the sample name and clustering results, and the vertical axis represents differentially expressed genes and clustering results. Red and blue represent highly and minimally expressed genes, respectively. (**f**) KEGG pathway enrichment analysis bubble plot. The abscissa is the ratio of the number of differential genes to the total number of differential genes, and the ordinate is the functional description. (**g**) KEGG pathway differential analysis histogram. The abscissa is the functional description, and the ordinate is –log10(padj), which represents the degree of difference in each pathway between the two groups.

In order to further mechanically explore the effects of different detachment processes on the physiological state of cells, we performed transcriptomic detection on cells detached with animal-based enzymes or animal-origin-free enzymes at 37 °C. The specific experimental process and group settings are shown in Figure 1. The FPKM (fragments per kilobase of transcript per million fragments mapped) box line plot of the sample distribution is shown in Figure 3a. The dispersion of gene expression level distribution per sample was small, and the overall gene expression was high. The principal component analysis (PCA) of total unique genes shows that genes in each treatment were homogenously expressed at principal component 1 (PC 1) with 26.91% variance (Figure S3a). The cluster diagram of the sequencing samples obtained according to gene expression is shown in Figure 3b. Extracting FPKM expression after the centralization and normalization of differential genes, performing a hierarchical cluster analysis, and plotting the cluster heat map for each differential grouping showed that there were significant differences in gene expression in the cells treated with animal-based enzymes or animal-origin-free enzymes (Figure 3c–e). In conclusion, we must further search for differentially expressed genes.

Next, we drew Venn diagrams of co-expression by comparing the gene expression 0 h, 8 h, and 24 h after digesting the cells with animal-based enzymes or animal-origin-free enzymes at 37 °C, as shown in Figure 3c (upper left, upper right). At the same time, the gene expression levels 0 h, 8 h, and 24 h after animal-origin-free enzymes at 4 °C were used to detach the cells at room temperature are shown in Figure 3c (bottom left). The results show that the expression levels of more than 12,300 genes remained unchanged during cell detachment, regardless of whether 37 °C animal-based enzymes, 37 °C animal-origin-free enzymes, or 4 °C animal-origin-free enzymes were used, as well as the control group, indicating that these genes do not change with the process or conditions of cellular detachment. In addition, the gene expression of the 0 h group detached with animal-based enzymes, 37 °C animal-origin-free enzymes, 4 °C animal-origin-free enzymes, and the nonenzyme-detached solution control group were analyzed for gene expression, and the Venn diagram of co-expression is shown in Figure 3c (bottom right). The results show that the expression levels of 12,642 genes were independent of the type of digestive solution used.

The differentially expressed genes (DEGs) were analyzed and the total, upregulated, and downregulated DEGs per group were enumerated (Table S1). A volcano map shows the overall distribution of the DEGs in both sample groups (Figure 3d). After software analysis, screening was performed with the criteria of log2 (FoldChange) > 0 and *p*-value < 0.05, and a total of 1237 differentially expressed genes with significant relationships were obtained, including 531 upregulated genes and 706 downregulated genes, as shown in Figure 3d (left). When the cells were harvested after digesting with animal-based enzymes or animal-origin-free enzymes at 37 °C for 8 h, there were differences in the expression levels of 3844 genes, of which 1944 were upregulated and 1900 were downregulated, as shown in Figure 3d (middle). When the cells were harvested after digesting with animal-based enzymes or animal-origin-free enzymes at 37 °C for 24 h, the differences in the expression levels of 4438 genes were detected, including 2012 upregulated genes and 2426 downregulated genes, as shown in Figure 3d (right). The results show that there were significant differences in the effects of animal-based enzymes and animal-origin-free enzymes on the physiological state of cells.

In order to further compare the effects of animal-based enzymes and animal-origin-free enzymes on gene expression levels, we used the sum of the differential genes of Vero cells (0 h group) and the control group to digest the animal-based enzymes and animal-origin-free enzymes at 37 °C; as for the gene list, the 0 h group, the 8 h group, and the 24 h group were detached with the two detachment solutions for cluster analysis. The heat map is shown in Figure 3e. The results show that, in the cluster analysis, the 37 °C animal-origin-free enzymes 0 h group (i.e., G2_0h) was the closest to the control group (i.e., Control), and the 37 °C animal-based enzymes 0 h group (i.e., G1_0h) was the farthest from the control group. The result indicates that the gene expression level of the 37 °C animal-origin-free enzymes 0 h group was the closest to the control group, and the difference between the 37 °C animal-based enzymes 0 h group and the control group was the largest. To sum up, during the detachment process, the animal-based enzymes had a greater effect on the expression level of cellular genes than the animal-origin-free enzymes.

In order to explore which physiological functions are involved in the genes affected by animal-based enzymes and animal-origin-free enzymes, we performed KEGG pathway enrichment analysis on the differentially expressed genes. The KEGG pathway enrichment bubble map is shown in Figure 3f, and the histogram of differences between KEGG pathways is shown in Figure 3g. The results show that the animal-based enzymes had the most significant effect on the oxidative phosphorylation pathway: a total of 53 genes in this pathway had significant changes in expression levels (Table S1), of which the COX17 gene expression level was significantly reduced. Since COX17 is a Cytochrome C Oxidase Copper Chaperone [35], COX17 plays an important role in the process of transporting Cu to the mitochondria. Therefore, animal-based enzymes may affect the normal physiological function of mitochondria.

### 3.3. Proteomic Analysis of Different Detachment Processes

**Figure 4:**
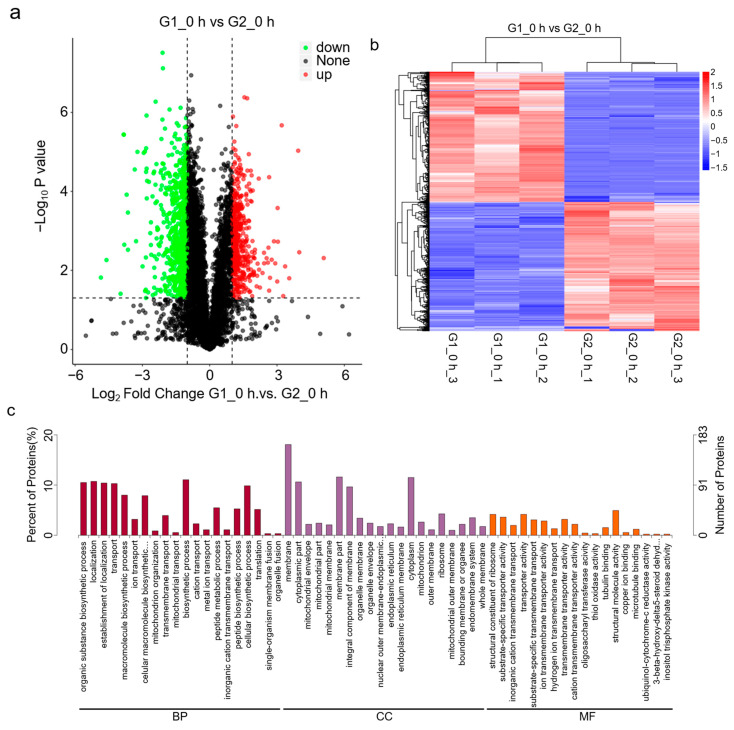

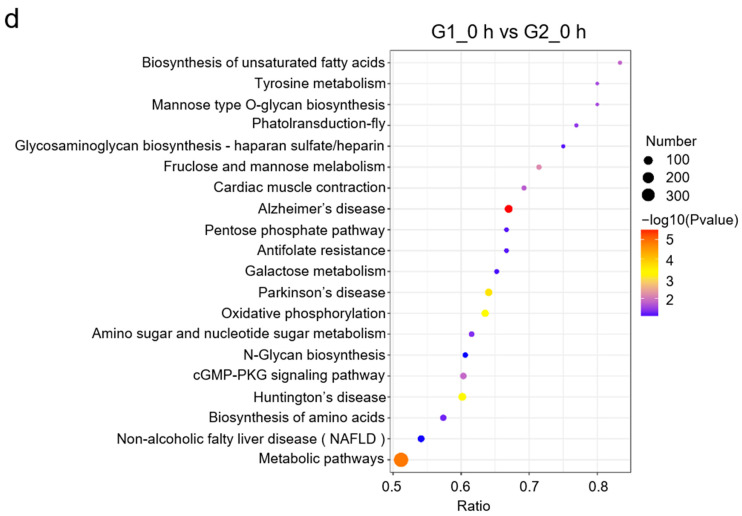
Proteomic analysis of animal-based enzymes and animal-origin-free enzymes. (**a**) Volcano plot of protein expression differences. G1 represents Group 1, which comprises 37 °C animal-based enzymes digesting Vero cells at 37 °C; G2 represents Group 2, which comprises 37 °C animal-origin-free enzymes digesting Vero cells at 37 °C. Green represents the weakly expressed proteins of G1_0h compared with G2_0h; red represents highly expressed proteins; black represents proteins with no significant difference in expression. (**b**) Differential clustering heatmap of protein expression. The horizontal axis represents the sample name and clustering results, and the vertical axis represents differentially expressed proteins and clustering results. Red and blue represent highly and weakly expressed proteins, respectively. (**c**) GO enrichment analysis histogram. The abscissa shows the functional description, and the ordinate shows the ratio of the number of differential proteins to the total number of differential proteins. CC (Cellular Component) stands for cellular component and is used to describe the subcellular structure, location, and macromolecular complexes, such as the nucleolus, telomeres, and complexes that recognize initiation; MF (molecular function) is used to describe the individual functions of genes and gene products, such as carbohydrate binding or ATP hydrolase activity, etc. BP (biological process) is used to describe the biological process in which the gene-encoded product participates, such as mitosis or purine metabolism. (**d**) KEGG pathway enrichment analysis bubble plot. The abscissa shows the ratio of the number of differential proteins to the total number of differential proteins, and the ordinate shows the functional description.

In order to explore the effects of animal-based enzymes and animal-origin-free enzymes on the protein levels of Vero cells, we treated cells with animal-based enzymes, animal-origin-free enzymes, and nonenzymatic digests at 37 °C, as shown in Figure 1. Proteomic detection was performed using the procedure shown. There were 1507 differentially expressed proteins caused by the detachment of Vero cells with animal-based enzymes and animal-origin-free enzymes, and the differential proteins were drawn as a volcano map as shown in Figure 4a, and as a differential protein heat map as shown in Figure 4b.

In order to explore which biological processes are involved in the proteins affected by animal-based enzymes and animal-origin-free enzymes, we performed GO enrichment analysis on the differential proteins of the two. With *p*-value ≤ 0.05 and FC ≥ 2 as the thresholds, the main biological functions of the differential proteins could be determined by GO significance analysis. The GO enrichment results of the differential proteins are shown in Figure 4c. The results show that the differential proteins caused by animal-based enzymes and animal-origin-free enzymes were mainly enriched in biological functions, such as the cell membrane, inner membrane, biosynthesis process, and transport.

In order to explore the physiological functions of proteins affected by animal-based enzymes and animal-origin-free enzymes, we performed KEGG pathway enrichment analysis on the differential proteins. Through KEGG pathway enrichment analysis, the most important biochemical metabolic pathways and signal transduction pathways involved in differential proteins can be determined. The KEGG pathway enrichment bubble map is shown in Figure 4d. The results show that the differential proteins caused by animal-based enzymes and animal-origin-free enzymes were mainly enriched in oxidative phosphorylation and metabolism pathways. This indicates that there were differences in the effects of animal-based enzymes and animal-origin-free enzymes on physiological functions, such as the oxidative phosphorylation of Vero cells, which were consistent with the above transcriptomic analysis results. Further, we observed that the animal-based enzymes caused a significant decrease in the protein levels of COX17 in the oxidative phosphorylation pathway, which corresponds to the decrease in the expression level of the COX17 gene in the previous transcriptomic test results, indicating that animal-based enzymes may regulate the mRNA and protein levels of COX17, which may further affect the normal physiological function of mitochondria.

### 3.4. Metabolomics Analysis of Different Detachment Processes

**Figure 5:**
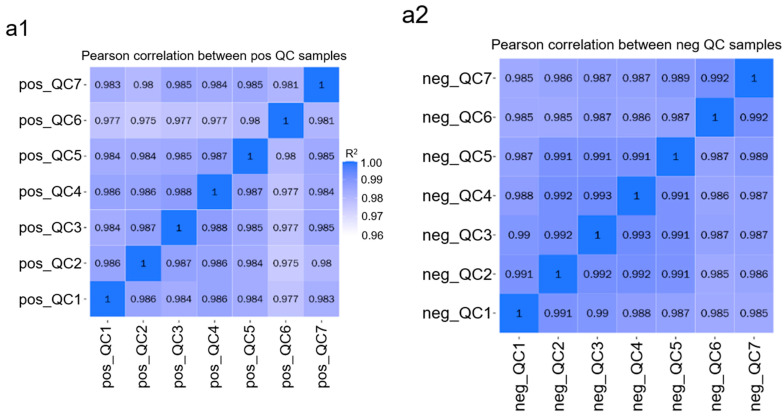

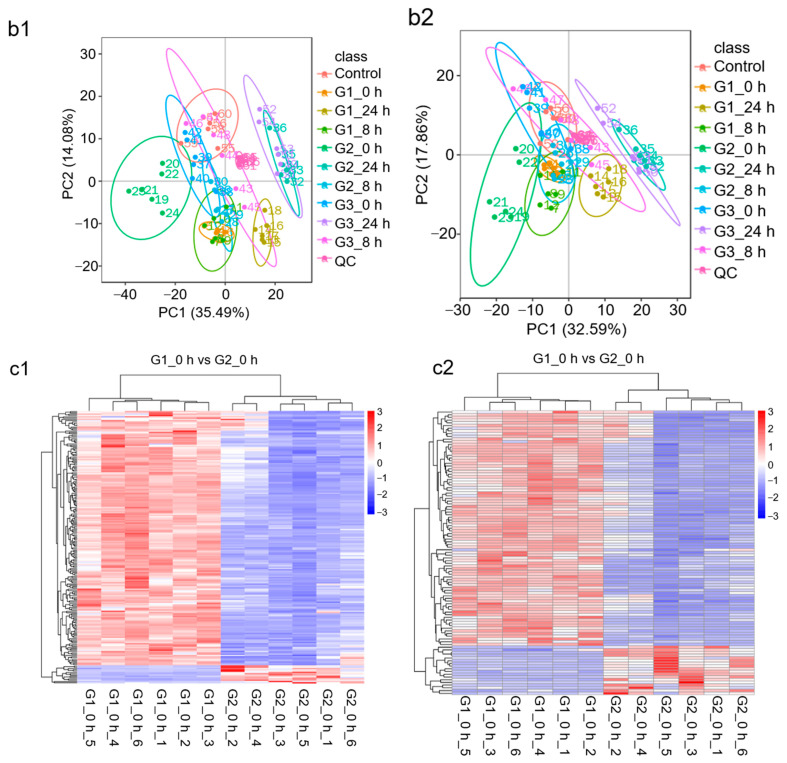

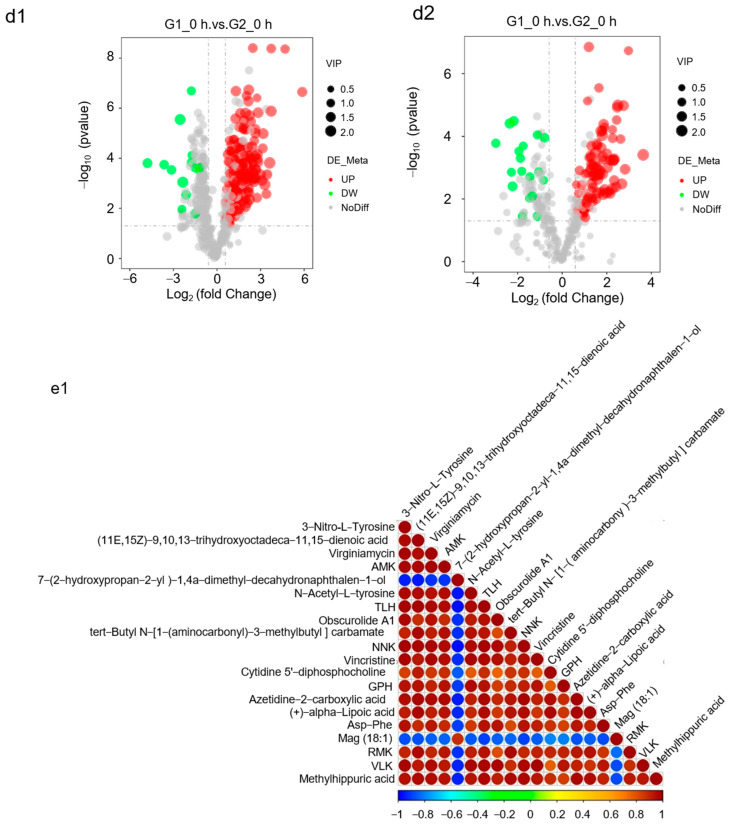

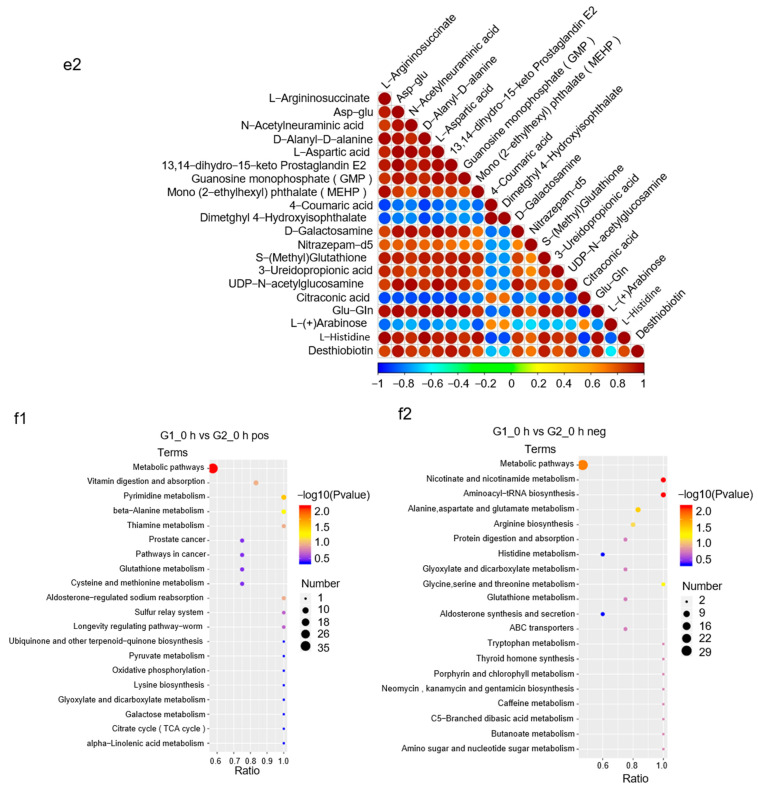
Metabolomics analysis of animal-based enzymes and animal-origin-free enzymes. (**a**) Correlation analysis diagram of quality control (QC) samples. Pearson correlation coefficient between QC samples was calculated based on the relative quantitative values of metabolites. (**a1**) is the Pearson correlation coefficient between seven groups of data QC samples in positive ion mode. (**a2**) is the Pearson correlation coefficient between seven groups of data QC samples in positive ion mode. Pearson correlation coefficient between seven groups of data QC samples. (**b**) PCA analysis diagram of total samples. The peaks extracted from all the experimental samples and QC samples were analyzed using PCA. In the figure, the abscissa PC1 and ordinate PC2 represent the scores of the first and second principal components, respectively; the scatter points of different colors represent samples in different experimental groups; and the ellipse represents the 95% confidence interval. (**b1**) is the PCA analysis diagram of the total sample in the positive ion mode, and (**b2**) is the PCA analysis diagram of the total sample in the negative ion mode. (**c**) Differential metabolite clustering heat map. Hierarchical clustering analysis was performed on the two groups of differential metabolites to obtain the differences in metabolic expression patterns between and within the two groups. The abscissa represents the sample name and clustering results, and the ordinate represents the differential metabolites and clustering results. Red and blue represent high and low expression metabolites, respectively. (**c1**) is the heat map of differential metabolites in positive ion mode, and (**c2**) is the heat map of differential metabolites in negative ion mode. (**d**) Volcanic map of differential metabolites. The abscissa represents the fold change of metabolites in different groups (log2(Fold Change)), and the ordinate represents the significant level of difference (–log10 (*p*-value)). Each point in the volcano diagram represents a metabolite, and the significantly upregulated metabolites are represented by red points and the significantly downregulated metabolites are represented by green points. The size of the dots represents the VIP value. (**d1**) is the volcano map of differential metabolites in positive ion mode, and (**d2**) is the volcano map of differential metabolites in negative ion mode. (**e**) Correlation analysis diagram of differential metabolites. Pearson’s correlation coefficient between all metabolites was calculated to analyze the correlation between each metabolite. When the linear relationship between the two metabolites is strengthened, the positive correlation tends to 1, and the negative correlation tends to –1. At the same time, the statistical test of significance was carried out on the correlation analysis of metabolites, and the threshold of significant correlation was selected as the significance level *p*-value < 0.05. (**e1**) is the correlation analysis diagram of differential metabolites in positive ion mode, and (**e2**) is the correlation analysis diagram of differential metabolites in negative ion mode. (**f**) Differential metabolite KEGG enrichment bubble diagram. According to the enrichment results of differential metabolites, the bubble chart of the KEGG pathway was drawn (only the top 20 results are shown). In the figure, the horizontal coordinate is x/y (the number of differential metabolites in the corresponding metabolic pathway/the number of total metabolites identified in the pathway). The higher the value, the higher the enrichment degree of differential metabolites in the pathway. The color of the dot represents the *p*-value of the hypergeometric test, and the smaller the value, the greater the reliability and statistical significance of the test. The size of the dot represents the number of differential metabolites in the corresponding pathway, and the larger the dot, the more differential metabolites in the pathway. (**f1**) is the KEGG enrichment bubble diagram of differential metabolites in positive ion mode, and (**f2**) is the KEGG enrichment bubble diagram of differential metabolites in negative ion mode.

In order to further explore the effects of animal-based enzymes and animal-origin-free enzymes on the metabolic level of Vero cells, cells treated with animal-based enzymes, animal-origin-free enzymes or enzyme-free digestive solution at 37 °C were subjected to metabolomics detection according to the process shown in Figure 2. The results show that the Pearson correlation coefficient between QC samples was calculated based on the relative quantitative values of metabolites [36]. In positive ion mode, the correlation of QC samples is shown in Figure 5a1; in negative ion mode, the correlation of QC samples is shown in Figure 5a2. PCA analysis was performed on the extracted peaks of all experimental samples and QC samples, as shown in Figure 5b1,b2. The above results show that the better the stability of the whole detection process, the higher the data quality.

The results of untargeted metabolomics technology show that a total of 615 positive-ion-mode metabolites and 351 negative-ion-mode metabolites were identified in 60 samples, including the control. Differential metabolites were screened according to the criteria of VIP > 1.0, FC > 1.5 or FC < 0.667, and *p*-value < 0.05. Based on the above data, hierarchical clustering analysis was performed on the differential metabolites obtained in each group to determine the differences in metabolic expression patterns between and within the two groups in the same comparison pair, as shown in Figure 5c1 (positive ion mode) and Figure 5c2 (negative ion mode).

Volcanic map results show that 210 positive-ion-mode metabolites were significantly different in the G1_0h vs. G2_0h comparison group, among which 196 metabolites were upregulated and 14 metabolites were downregulated (Figure 5d1). There were 105 negative-ion-mode metabolites that were significantly different, with 87 metabolites upregulated and 18 metabolites downregulated (Figure 5d2). See Figure 5e1,e2 for correlations of metabolites with significant differences between positive/negative ion mode animal-based enzyme and animal-origin-free enzyme groups. In order to explore the physiological functions of metabolites affected by animal-based enzymes and animal-origin-free enzymes, KEGG pathway enrichment analysis was performed on the differential metabolites of animal-based enzymes and animal-origin-free enzymes. Through KEGG pathway enrichment analysis, the major biochemical metabolic pathways and signal transduction pathways involved in differential metabolites could be determined. The most significant 20 pathways were selected to draw bubble charts, as shown in Figure 5f1 (positive ion mode) and Figure 5f2 (negative ion mode).

The results show that, in positive ion mode, metabolic pathways, such as pyrimidine metabolism, beta-alanine metabolism, and glutathione metabolism were mainly involved, while in negative ion mode, nicotinate and nicotinamide metabolism, aminoacyl-tRNA biosynthesis, alanine, aspartate, and glutamate metabolism, glutathione metabolism, and other metabolic pathways were mainly involved. We also found that the levels of spermine and spermidine metabolites in glutathione metabolism were significantly reduced under positive ion mode. In conclusion, animal-based enzymes may affect cell glutathione metabolism by downregulating the levels of spermine and spermidine metabolites.

### 3.5. Combined Metabolomics and Transcriptomic Analysis

The genes with significant differences obtained by transcriptome analysis and metabolites with significant differences obtained by metabolomics analysis were co-enriched to obtain the corresponding metabolic pathway map. In the KEGG pathway diagram, circles represent metabolites; blue circles represent downregulated differential metabolites, and yellow circles represent upregulated differential metabolites. The boxes represent genes or proteins: green boxes represent genes whose expression levels are differentially downregulated, and red boxes represent genes whose expression levels are differentially upregulated.

**Figure 6:**
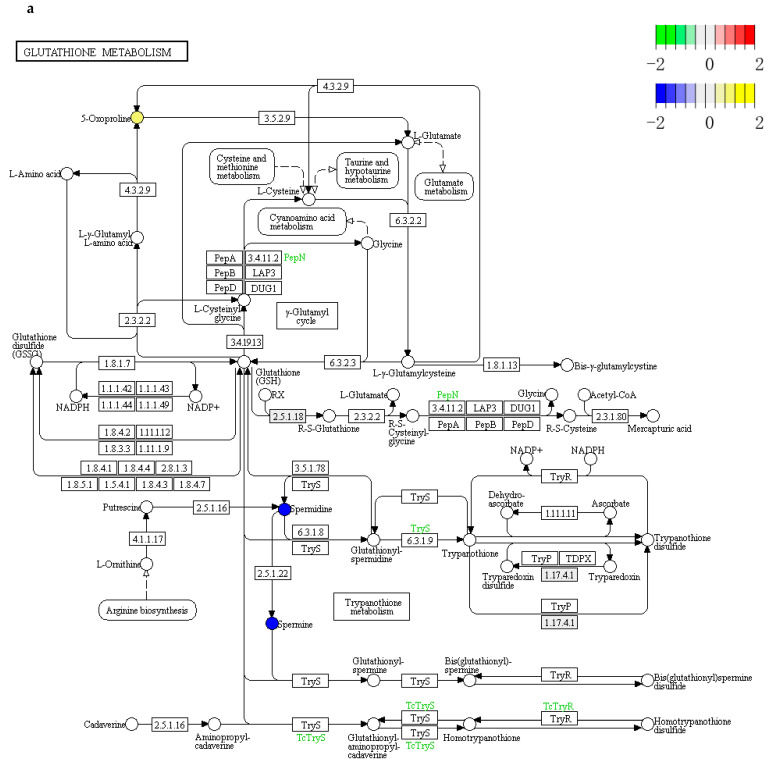

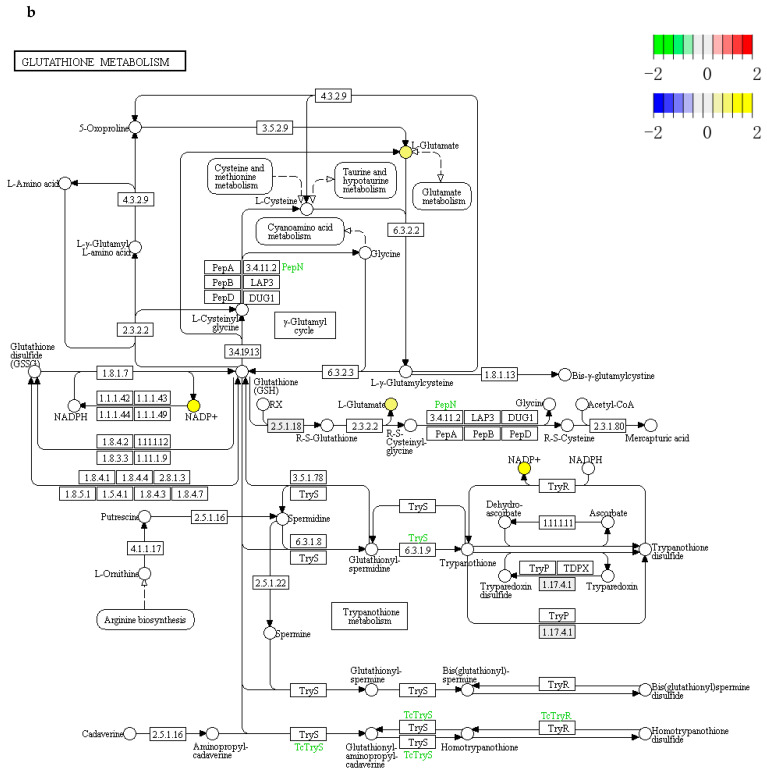
Combined metabolome and transcriptome analysis. (**a**) PathView enrichment pathway diagram (positive ion mode). According to the enrichment results, the metabolic pathways enriched in both metabolomics and transcriptomics are shown as pathway maps. In the KEGG pathway diagram, circles represent metabolites; blue circles represent downregulated differential metabolites, and yellow circles represent upregulated differential metabolites. The boxes represent genes or proteins, where the green boxes represent genes whose expression levels are differentially downregulated and the red boxes represent genes whose expression levels are differentially upregulated. (**b**) PathView enrichment pathway diagram (negative ion mode).

We found that the 37 °C animal-based enzymes 0 h group (G1_0h) versus the 37 °C animal-origin-free enzymes 0 h group (G1_0h) was involved in glutathione metabolism in Vero cells (Figure 6a,b). In positive ion mode, the cellular metabolite 5-hydroxyproline was significantly upregulated, and the metabolites spermine and spermidine were significantly downregulated (Figure 6a). In negative ion mode, the cellular metabolites nicotinamide adenine dinucleotide phosphate and L-glutamate were significantly upregulated (Figure 6b). The differentially downregulated genes were *PepN*, *TryS*, *TcTryS*, and *TcTryR*. In conclusion, the results of combined metabolome and transcriptome analysis further confirm that animal-based enzymes may affect the metabolism of glutathione by downregulating the levels of spermine and spermidine metabolites, and thus affect the normal physiological state of Vero cells.

## 4. Discussion

In recent years, the scale of cell-culture-based production of biologicals in the biopharmaceutical industry has continued to expand. For example, the value of >USD 100 billion in revenue per year generated by antibodies and similar molecules from CHO cells [37,38]. There is large demand for Vero cell substrates in the production of five billion doses of inactivated SARS-CoV-2 vaccine [17,39,40]. However, the effects of cell detachment technology on downstream target products and the mechanisms of cell physiological response during detachment are rarely reported. The use of animal-based enzymes to dissociate cells from the culture plate/bottle wall is the most common method in the traditional culture of adherent cells. However, there is no room for efficiency improvement in the study of detachment technology of animal-based enzymes extracted from natural biogenic sources. In recent years, emerging animal-origin-free engineering restructuring enzymes have also been widely used, mainly through pyrolysis lysine and arginine at the end of C-peptide bonds [23,24,25]. In order to improve the efficiency of cell substrate expansion cultures and production in biopharmaceutical processes, we systematically studied the mechanism of action of animal-based enzymes and animal-origin-free enzymes in the process of cell detachment and their effects on the cell physiological state by means of multi-omics technology.

In our study, we found that different detachment conditions had no significant effect on Vero cell viability, the cluster formation rate, cell growth and proliferation, or glucose metabolism. However, animal-based enzymes are more likely to cause cell apoptosis than animal-origin-free enzymes. Moreover, animal-based enzymes had a greater effect on cell gene expression levels than animal-origin-free enzymes during detachment. Proteomic results show that the differential proteins caused by animal-based enzymes and animal-origin-free enzymes were mainly enriched in steroid biosynthesis, oxidative phosphorylation, programmed necrosis, ribosome, senescence, metabolism, and other pathways. The results of combined metabolome and transcriptome analysis show that the differential metabolites and gene enrichment caused by animal-based enzymes and animal-origin-free enzymes were mainly involved in glutathione metabolism in Vero cells. In positive ion mode, the cell metabolite 5-hydroxyproline was significantly upregulated, and the metabolites spermine and spermidine were significantly downregulated.

We found that animal-based enzymes and animal-origin-free enzymes appeared to have little effect on other indicators of cell status, except for the apoptotic indicators, which are commonly used in conventional detachment processes. However, using multi-omics technology, we found that animal-based enzymes had a greater impact on transcription, metabolism, proteins, and other aspects during cell detachment than animal-origin-free enzymes. This indicates that the commonly used indicators of traditional cell detachment technology cannot fully reflect the true state of cells, and some microlevel change indicators need to be identified and discovered.

We found that the protein COX17 and the metabolites spermine and spermidine may be biomarkers to evaluate the cell passage process at the microlevel. COX17 is an important protein for cytochrome C oxidase assembly in mitochondria [41]. In the oxidase complex, COX17 provides copper ions for the formation of CuA and CuB sites [42]. In the cytoplasm, absorbed copper is transported by the copper chaperone COX17 to the mitochondria for metallization of cytochrome C oxidase (CcO) and enters the circulation through pumping by copper ATPase7a (ATP7a) located in the trans-Golgi network (TGN) [43]. The animal-based enzymes downregulated COX17 expression more than animal-origin-free enzymes, which undoubtedly disrupted this balance. We conclude that, compared with animal-origin-free enzymes, animal-based enzymes could change the expression level of oxidative stress-related biomarkers and activate mitochondria-induced apoptosis, and the toxic effect was mainly a result of the release of ion Cu^2+^.

Spermine and spermidine are essential cellular components that play important roles in cell proliferation, transformation, differentiation, apoptosis, protection from oxidative damage, regulation of ion channels, and the stabilization of important cellular components, such as the cell membrane and chromatin structure [44,45,46,47,48]. We found that animal-based enzymes significantly decreased spermidine and spermine levels compared with animal-origin-free enzymes, which further promoted the apoptosis of Vero cells.

Microscopic changes in cellular components, such as the COX17 protein, spermidine, and spermine metabolites may affect the state of the cell, thereby affecting the expansion of the virus, and ultimately the quality of the vaccine product.

## 5. Conclusions

Our study established the optimal detachment process of Vero cells by deeply characterizing the cell subculture process using multi-omics tools. We found new biomarkers, COX17, spermine, and spermidine, which may help us to evaluate the cell passage process. More refined evaluation tools are of great significance for cell-scale preparation.

In future research, animal-based enzymes, animal-origin-free enzymes, and the Vero cell passage process under detachment conditions will be further compared in cell factories or large-capacity fermenters combined with the operation parameters of process equipment, and the state of Vero cells will be actively monitored possibly by using evaluation tools, such as COX17, spermine, and spermidine. By that time, the large-scale production of Vero cells in a minimal-damage state might be used to better guide the industrial production of biomedicines.

## Supplementary Materials

The following supporting information can be downloaded at: https://www.mdpi.com/article/10.3390/cells11213396/s1, Figure S1: Animal-based enzymes had no significant effect on cell viability, aggregation rate, cell growth and proliferation, or glucose metabolism compared with animal-origin-free enzymes; Figure S2: Animal-based enzymes induce cell apoptosis compared with animal-origin-free enzymes at 25 °C; Figure S3: Analysis of mRNA expression level; Figure S4: Analysis of protein expression level; Figure S5: Combined analysis of transcriptomics and proteomics; Table S1: List of differentially expressed RNAs, proteins, and metabolites.
