# Supplementary material for: Integrated Multi-Omic Characterization of the Detachment Process of Adherent Vero Cells with Animal-Based and Animal-Origin-Free Enzymes
Source: Cells. 2022 Oct 27;11(21):3396. doi: 10.3390/cells11213396 (PMC9656133; doi:10.3390/cells11213396)
Supplement: Supplementary file 1 [file cells-11-03396-s001.zip › cells-1927745 Supplementary figures.pdf]

cells MDPI

# Supplementary Figures

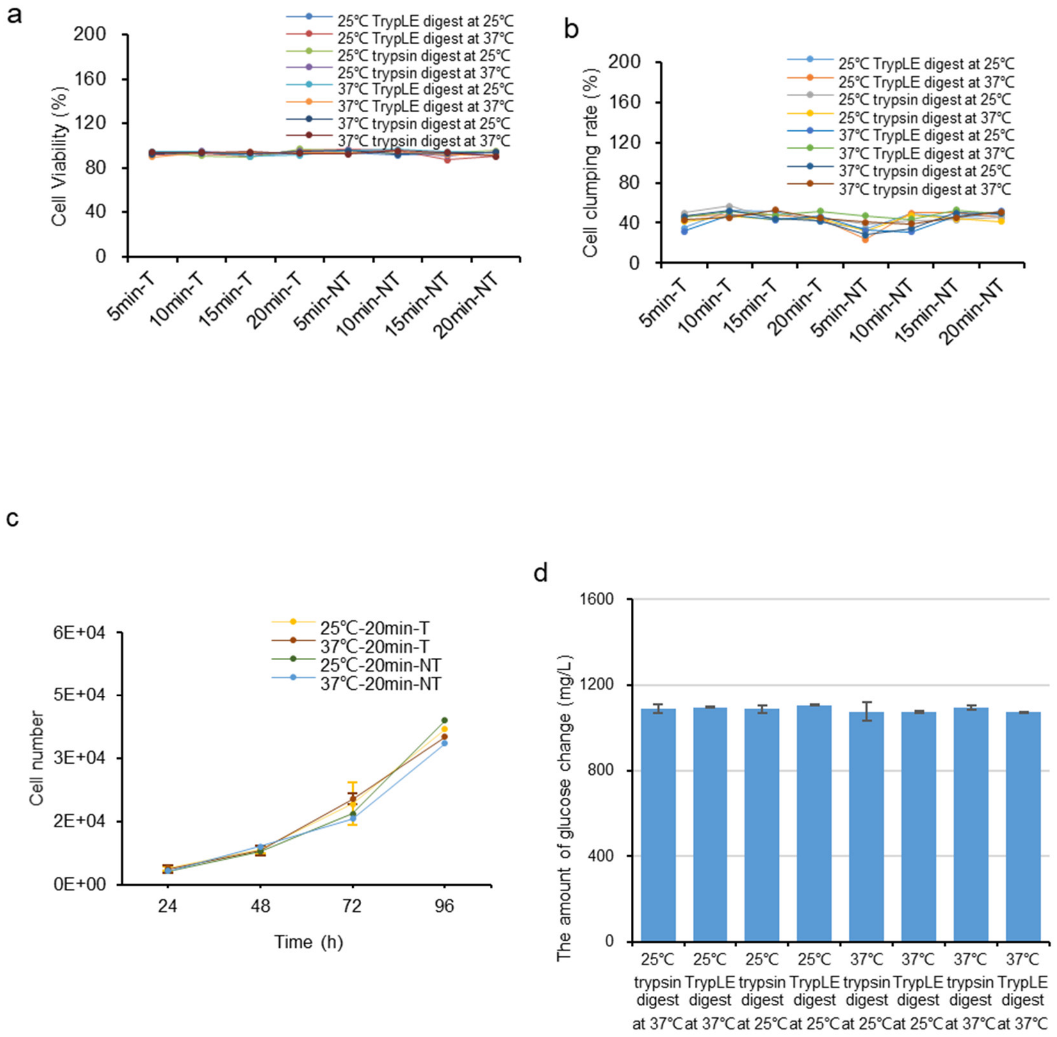


**Figure S1.** Animal-based enzymes had no significant effect on cell viability, clustering rate, cell growth and proliferation, or glucose metabolism compared with animal origin-free enzymes. (**a**) Vero cells were plated into 24-well plates, 2 × 10$^5$ cells per well, and after culturing in a 37 °C cell incubator for 12–24 h, cells were treated with different digestion conditions, and cell viability was directly detected. T represents the cells treated with animal-based enzymes (trypsin) or animal origin-free enzymes (TrypLE), and the digestion reaction was terminated with cell growth solution containing 10% FBS; NT represents the cells treated with animal-based enzymes or animal origin-free enzymes, and the digestion reaction was not terminated with cell growth solution containing 10% FBS. The time marked in the figure is the time of cell digestion. (**b**) Vero cells were plated into 24-well plates, 2 × 10$^5$ cells per well, and after culturing in a 37 °C cell incubator for 12–24 h, cells were treated with different digestion conditions, and clustering rate was directly detected. T represents the cells treated with animal-based enzymes (trypsin) or animal origin-free enzymes (TrypLE), and the digestion reaction was terminated with cell growth solution containing 10% FBS; NT represents the cells treated with animal-based enzymes or animal origin-free enzymes, and the digestion reaction was not terminated with cell growth solution containing 10% FBS. The time marked in the figure is the time of cell digestion. (**c**) 5 × 10$^5$ cells were plated in each well of a 6-well plate, and after culturing for 12–24 h, Vero cells were digested with animal origin-free enzymes (TrypLE) at 25 °C/37 °C for 20 min. Then, the cells were counted, and the cells were plated into 96-well plates with 5000 cells per well. After culturing in the 96-well plates for 24, 48, 72, and 96 h, the number of cells was detected respectively. In the figure, T represents that after the cells were treated with animal origin-free enzymes, the digestion reaction was terminated with a cell growth solution containing 10% FBS;

NT stands for animal origin-free enzymes treatment of cells, the digestion reaction is not terminated with cell growth solution containing 10% FBS. (**d**) Plate Vero cells into 24-well plates, 2 × $10^5$ cells per well, and after culturing in a 37 °C cell incubator for 12–24 h, take 1 mL of growth solution from each well to detect the glucose content, which is the glucose metabolism of the cells before digestion. Then, after digesting the cells for 20 min by different digestion processes in each well, they were spread into corresponding new 24-well plates, cultured in a 37 °C cell incubator for 2 h, and 1 mL of supernatant growth solution was taken from each well to detect the glucose content, which is the amount of glucose metabolized after digestion. The difference between the post-digestion glucose metabolism and the pre-digestion glucose metabolism is calculated as the changed glucose metabolism. 3-well replicates were performed for each digestion process condition.

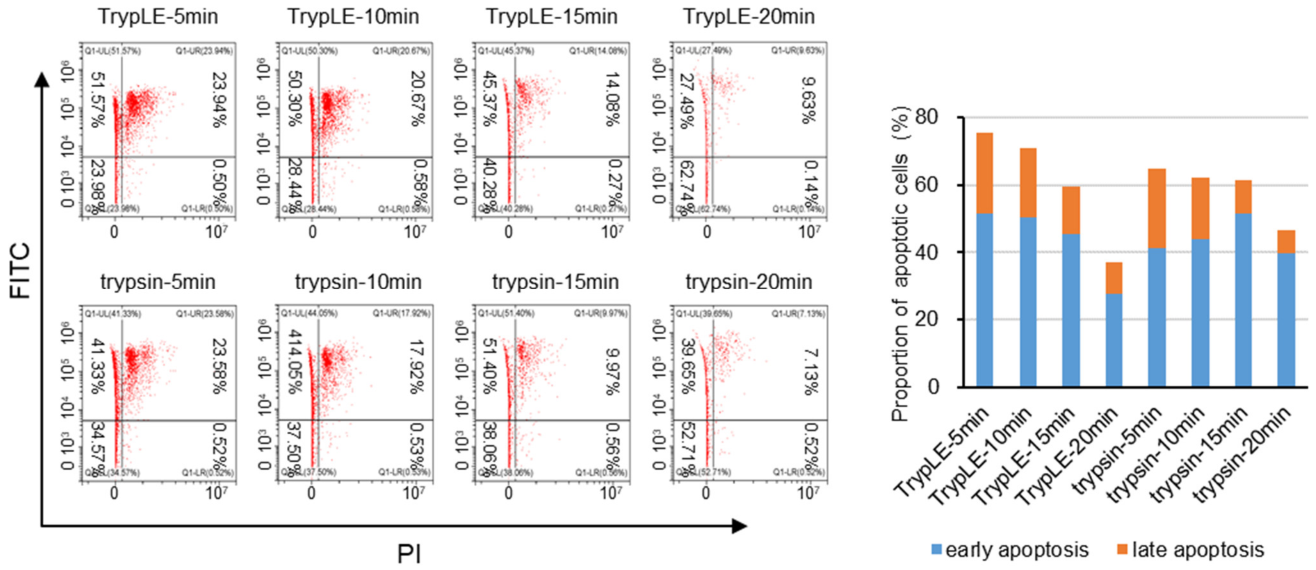


**Figure S2.** Animal-based enzymes induce cell apoptosis compared with animal origin-free enzymes at 25 °C. 5 × $10^5$ cells were plated in each well of a 6-well plate, and after culturing for 12–24 h, the cells were digested with animal-based enzymes (trypsin) or animal origin-free enzymes (TrypLE) at 25 °C for 5, 10, 15, and 20 min, and FITC/PI dual-color labeling flow cytometry was performed.

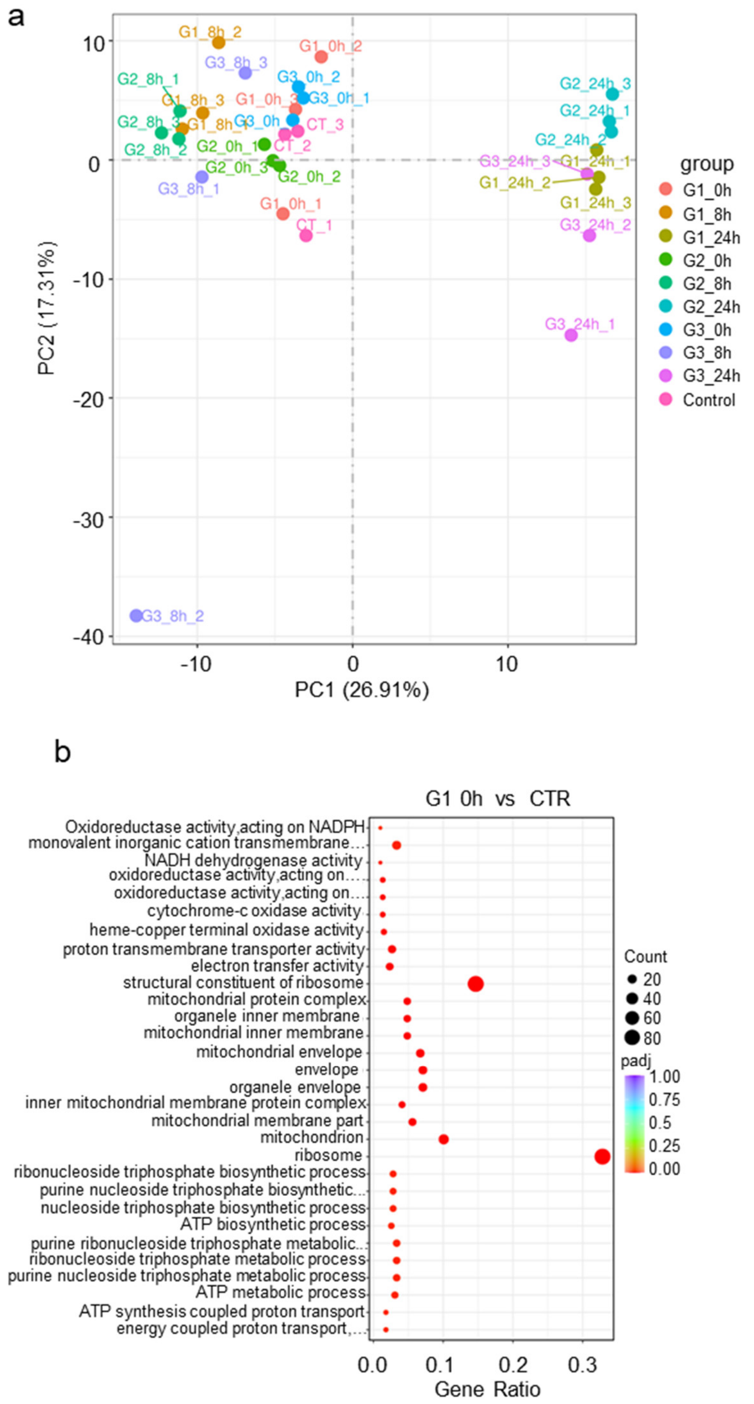
a
PC1 (26.91%)
PC2 (17.31%)
group
G1_0h
G1_8h
G1_24h
G2_0h
G2_8h
G2_24h
G3_0h
G3_8h
G3_24h
Control
b
G1 0h vs CTR
Oxidoreductase activity,acting on NADPH
monovalent inorganic cation transmembrane...
NADH dehydrogenase activity
oxidoreductase activity,acting on...
oxidoreductase activity,acting on...
cytochrome-c oxidase activity
heme-copper terminal oxidase activity
proton transmembrane transporter activity
electron transfer activity
structural constituent of ribosome
mitochondrial protein complex
organele inner membrane
mitochondrial inner membrane
mitochondrial envelope
envelope
organele envelope
inner mitochondrial membrane protein complex
mitochondrial membrane part
mitochondrion
ribosome
ribonucleoside triphosphate biosynthetic process
purine nucleoside triphosphate biosynthetic...
nucleoside triphosphate biosynthetic process
ATP biosynthetic process
purine ribonucleoside triphosphate metabolic...
ribonucleoside triphosphate metabolic process
purine nucleoside triphosphate metabolic process
ATP metabolic process
ATP synthesis coupled proton transport
energy coupled proton transport,...
Gene Ratio
Count
padj

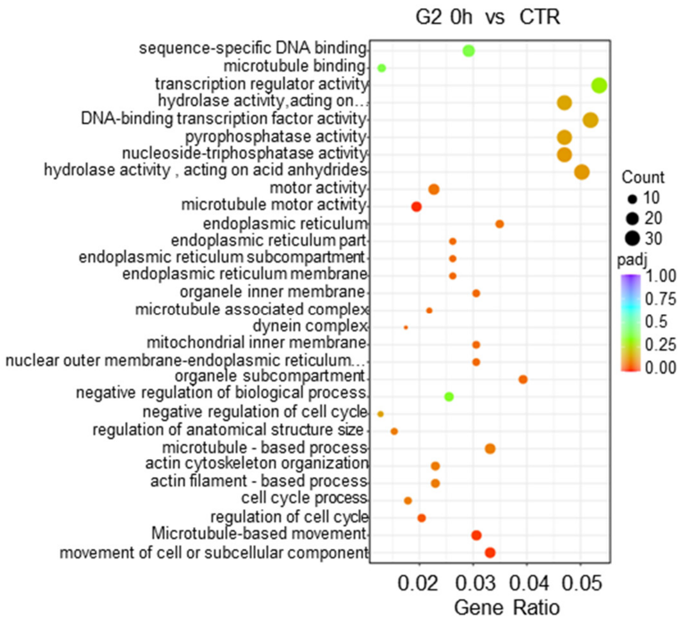


**Figure S3.** Analysis of mRNA expression level. (**a**) PCA for RNA-seq. The points represent different samples with three biological replicates. (**b**) GO enrichment analysis bubble plot. The abscissa is the ratio of the number of differential genes to the total number of differential genes, and the ordinate is the functional description. The depth of the node color represents the degree of enrichment. The darker the color, the higher the degree of enrichment. Each node displays the name of the TERM and the padj of the enrichment analysis. The up one represents GO enrichment analysis of G1 0 h group compared with control. The down one represents GO enrichment analysis of G2 0 h group compared with control.

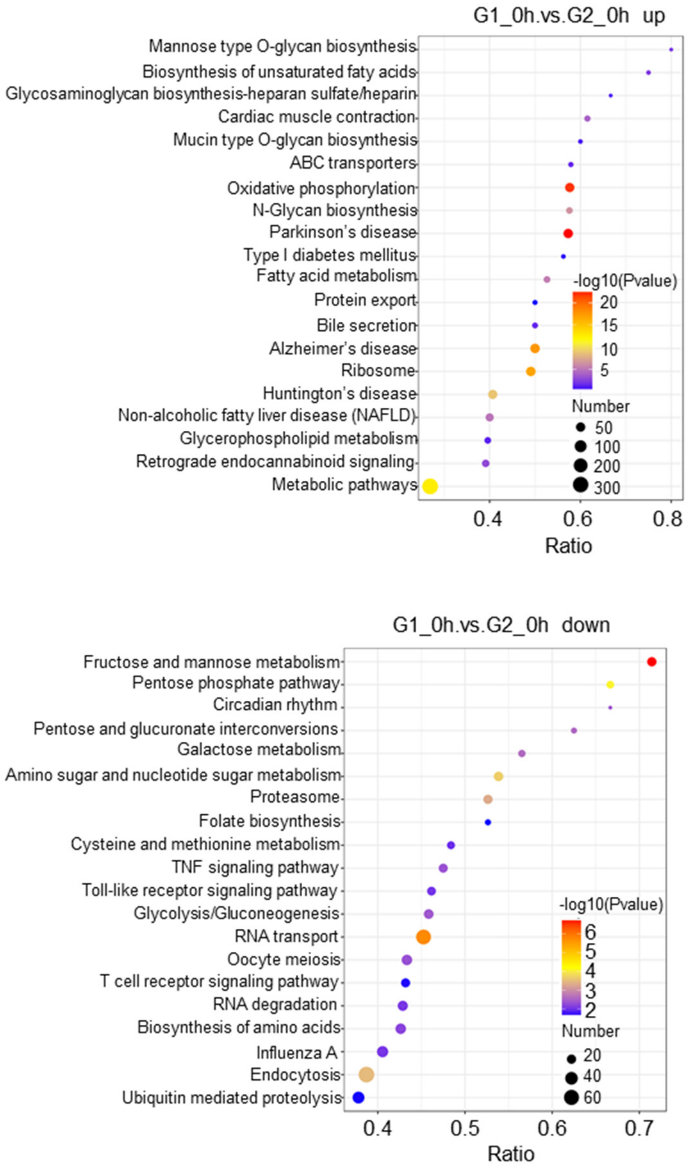


**Figure S4.** Analysis of protein expression level. KEGG pathway enrichment analysis bubble plot. The abscissa is the ratio of the number of differential proteins to the total number of differential proteins, and the ordinate is the functional description. The up one represents up-regulated proteins in cells treated with animal-based enzymes compared with animal origin-free enzymes, while the down one stands for down-regulated proteins in cells treated with animal-based enzymes compared with animal origin-free enzymes.

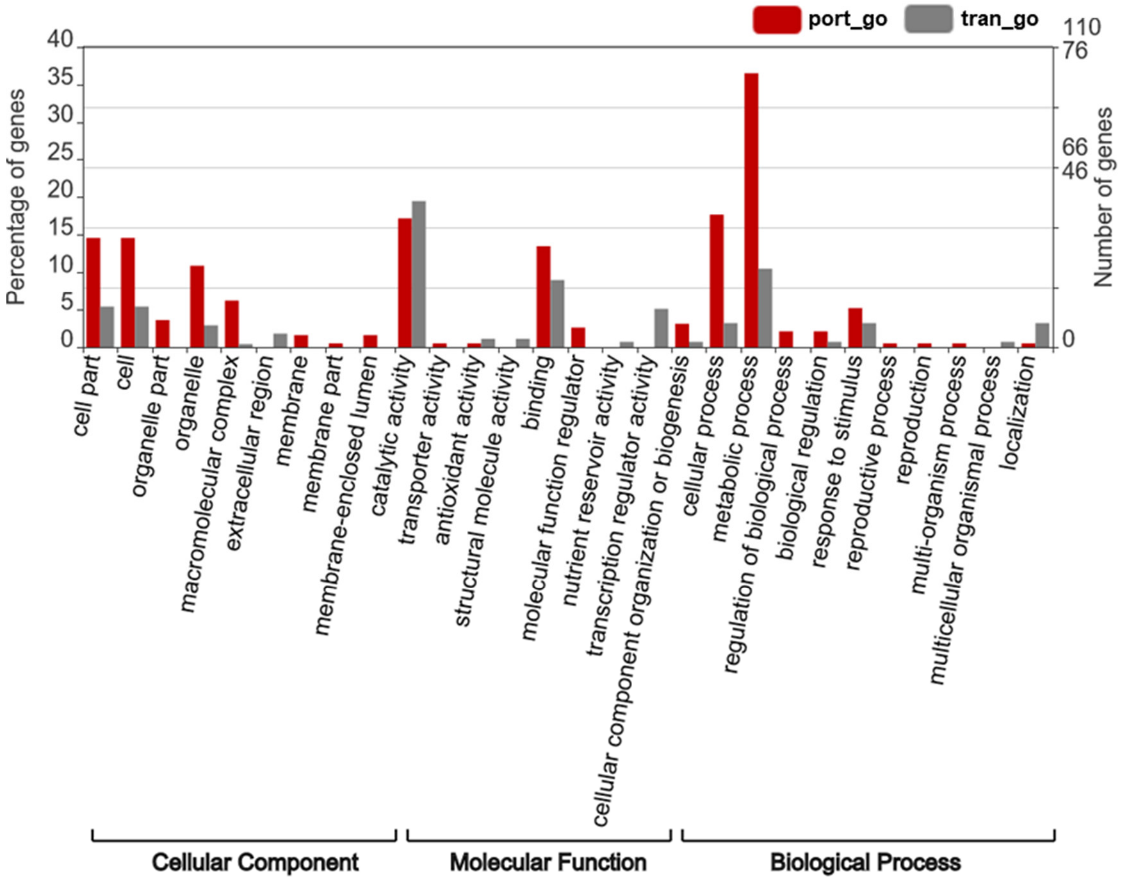


**Figure S5.** Combined analysis of transcriptomics and proteomics. GO enrichment analysis histogram. The abscissa is the functional description, and the ordinate is the ratio of the number of differential proteins to the total number of differential proteins. CC (Cellular Component) stands for cellular component and is used to describe subcellular structure, location and macromolecular complexes, such as nucleolus, telomeres and complexes that recognize initiation; MF (Molecular Function) stands for molecular function and is used to describe The individual functions of genes and gene products, such as carbohydrate binding or ATP hydrolase activity, etc.; BP (Biological Process) represents a biological process, which is used to describe the biological process in which the gene-encoded product participates, such as mitosis or purine metabolism.
